# Dominant Fecal Microbiota in Newly Diagnosed Untreated Inflammatory Bowel Disease Patients

**DOI:** 10.1155/2013/636785

**Published:** 2013-11-17

**Authors:** Lill Therese Thorkildsen, Felix Chinweije Nwosu, Ekaterina Avershina, Petr Ricanek, Gøri Perminow, Stephan Brackmann, Morten H. Vatn, Knut Rudi

**Affiliations:** ^1^Hedmark University College, 2306 Hamar, Norway; ^2^Department of Chemistry, Biotechnology and Food Science, Norwegian University for Life Sciences, 1432 Ås, Norway; ^3^Department of Gastroenterology, Akershus University Hospital, 1478 Lørenskog, Norway; ^4^Department of Clinical Molecular Biology and Laboratory Sciences (EpiGen), Division of Medicine, Akershus University Hospital, University of Oslo, 0316 Oslo, Norway; ^5^Pediatric Department, Oslo University Hospital, Ullevål, 0450 Oslo, Norway; ^6^Medical Clinic, Oslo University Hospital, Rikshospitalet, 0372 Oslo, Norway

## Abstract

Our knowledge about the microbiota associated with the onset of IBD is limited. The aim of our study was to investigate the correlation between IBD and the fecal microbiota for early diagnosed untreated patients. The fecal samples used were a part of the Inflammatory Bowel South-Eastern Norway II (IBSEN II) study and were collected from CD patients (*n* = 30), UC patients (*n* = 33), unclassified IBD (IBDU) patients (*n* = 3), and from a control group (*n* = 34). The bacteria associated with the fecal samples were analyzed using a direct 16S rRNA gene-sequencing approach combined with a multivariate curve resolution (MCR) analysis. In addition, a 16S rRNA gene clone library was prepared for the construction of bacteria-specific gene-targeted single nucleotide primer extension (SNuPE) probes. The MCR analysis resulted in the recovery of five pure components of the dominant bacteria present: *Escherichia/Shigella*, *Faecalibacterium*, *Bacteroides*, and two components of unclassified Clostridiales. *Escherichia/Shigella* was found to be significantly increased in CD patients compared to control subjects, and *Faecalibacterium* was found to be significantly reduced in CD patients compared to both UC patients and control subjects. Furthermore, a SNuPE probe specific for *Escherichia/Shigella* showed a significant overrepresentation of *Escherichia/Shigella* in CD patients compared to control subjects. In conclusion, samples from CD patients exhibited an increase in *Escherichia/Shigella* and a decrease in *Faecalibacterium* indicating that the onset of the disease is associated with an increase in proinflammatory and a decrease in anti-inflammatory bacteria.

## 1. Introduction

The gut microbiota has the potential to exert both pro- and anti-inflammatory responses [1–3]. The gut microbiota is also supposed to be an epigenetic factor modifying the pathogenesis of extraintestinal disorders, including type I diabetes [4], obesity [5], atopic disorders such as asthma and eczema [6], and a contributing factor in the pathogenesis of inflammatory bowels disease (IBD) [7]. Knowledge of the composition of the intestinal microbiota, therefore, is vital to our understanding of which groups of bacteria are of importance in maintaining gut health or promoting disease.

The two major forms of IBD are ulcerative colitis (UC) and Crohn's disease (CD) [8, 9]. The etiology of IBD is complex and the causes are not yet fully understood. The pathogenesis of IBD involves interactions between the intestinal microbiota, the immune system, and epithelial cells. In addition, genetic and environmental factors modify this interplay towards or away from disease [10]. While these results are not conclusive, environmental factors do seem to influence the development of IBD. 

Intestinal microorganisms have been implicated in the pathogenesis of IBD, with abnormal interactions between the host and either pathogens or commensal bacteria. Altered microbial composition and function result in increased immune stimulation, epithelial dysfunction, or enhanced mucosal permeability [11]. Studies have revealed that experimental colitis does not develop in animals when they are kept in a germ-free environment, suggesting that normal mucosal microbiota is required to initiate or maintain an inflammatory process [12]. The link between enteric bacteria and mucosal inflammation is also strengthened by the role of the CD susceptibility gene, NOD2/CARD15, in bacterial peptidoglycan recognition [13]. Moreover, IBD especially occurs in the colon and distal ileum, which contain the highest intestinal bacterial concentrations. Furthermore, antibiotics can reduce inflammation [14] while diversion of the fecal stream can prevent recurrence in CD [15].

In most previous studies, where samples from IBD patients have been under study, the samples have often been from long-term patients who have already received treatment for their medical conditions. Such treatment can lead to modifications of the fecal microbiota that subsequently influence the analytical outcome. It has been proposed that analysis of gastrointestinal microbiota in established IBD more accurately reflects changes associated with chronic disease, and as such should not be extrapolated to the onset of disease [16]. In the current study, however, fecal samples were collected from newly diagnosed IBD patients that had not yet received treatment for their disease. Hence, the sample set used in this study is unique as it describes the fecal microbiota at the onset of disease in untreated IBD patients. 

The aim of the current study was to determine any correlation of fecal microbiota composition to IBD patients (both CD and UC) by comparing fecal samples of IBD patients to non-IBD control subjects, in an attempt to study the relationship between microbiota and established inflammation. In order to achieve this aim, we used direct sequencing of 16S rRNA gene sequences amplified from bacterial DNA extracted from the fecal samples [17, 18], in addition to a validation of our findings using a targeted probe approach [19].

## 2. Materials and Methods

**Figure 1:**
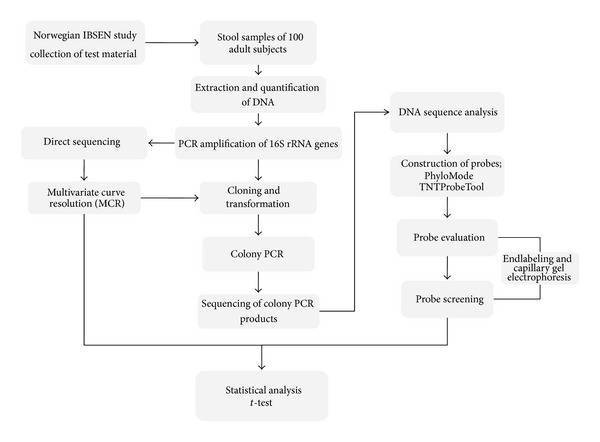
Schematic outline of the methodology.

A schematic outline of the methodology used in this work is given in Figure 1.

### 2.1. Subjects and Study Design

The stool samples used in the current study were from patients with newly diagnosed untreated IBD, and non-IBD patients were used as controls (Table 1). These samples were part of the Inflammatory Bowel South-Eastern Norway II study (the IBSEN II study) and were provided by Akershus University Hospital (Ahus) during 2005–2007. The subjects included were patients suspected to have IBD on the basis of a set of predefined symptoms, including abdominal pain, diarrhea, and/or blood in the stools for more than 10 days. An IBD diagnosis was based upon endoscopic and histologic findings. The IBD diagnosed patients were classified as CD, UC, or IBDU (IBD unclassified) based on ileocolonoscopy with addition of histology for each segment of the bowel, according to the Lennard-Jones criteria [20] and the Vienna classification [21]. Patients with IBD that could not be attributed to CD or UC were classified as IBD unclassified (IBDU). 

Subjects who did not meet the diagnostic criteria for IBD and who displayed no evidence of infection or other pathology in the gut were included as a symptomatic non-IBD control group. Subjects with infection of pathogenic gut bacteria, microscopic colitis, or cancer were excluded from the IBSEN II study, both for cases and controls [22]. 

Of the 30 CD patients, four (13%) showed ileal disease (L1), 17 (57%) colonic disease (L2), and 9 (30%) ileocolonic disease (L3). A fistula was found in two (7%) and a stenosis in in four (13%) CD patients. Twenty-four had a nonstricturing/nonpenetrating behaviour. Most of these patients had a mild clinical disease with a median Harvey Bradshaw Index of five (range 0 to 29). 

Among the 33 patients with UC, 17 (52%) had total or extensive colitis, four (12%) had left sided, and 12 (36%) proctitis. Also in the group of UC patients, the clinical disease was relatively mild with a median Simple Clinical Colitis Activity Index of four (range 0 to 14).

In total, ninety-nine patients from the IBSEN II study were included in this present study, ages ranging from 16 to 60 years. Out of the hundred patient samples, 33 were diagnosed with UC, while 30 were diagnosed with CD. In addition, 3 patients were diagnosed with unclassified IBD (IBDU). Samples of 34 subjects were in the non-IBD control group. Extraintestinal manifestations were found in three (10%), three (9%), and two (67%) of the patients with CD, UC and IBDU, respectively. 

All CD, UC, and IBDU patients were included in the primary stage of treatment naive active disease.

Among the included patients, four (6%) IBD patients had been using antibiotics within one week and five patients (8%) within one month prior to stool sampling. Among the non-IBD controls, none had used antibiotics within one week, but three (9%) within one month prior to stool sampling.

### 2.2. Stool Samples

Patients were informed to collect stool before cleansing and received equipment for collection. Samples were kept cooled by the patients in a refrigerator and delivered at the day of the endoscopic examination. The samples were then deep frozen at −80°C the same day. Only a few patients failed to deliver a stool sample at inclusion. 

### 2.3. DNA Extraction

DNA was extracted using the QIAGEN QIAamp DNA Stool Mini Kit (Qiagen, Hilden, Germany). The purification of the DNA from the stool samples was done according to the manufacturer's instructions. The stool samples were stored at −80°C before approximately 200 mg of the samples was used for the DNA extraction. The samples were lysed in 1.6 mL ASL buffer (Qiagen) with a bead-beating step of 2 minutes at 20 Hz in order to ensure maximum yield. The samples were then heated at 95°C for 5 minutes for further lysis. After cooling in room temperature the samples were vortexed before being centrifuged at 17 g for 1 minute to pellet stool particles. One InhibitEXtablet was added to 1.4 mL of the supernatant. The samples were incubated for one minute in room temperature to allow inhibitors to adsorb the InhibitEX matrix. The samples were then centrifuged at 17 g for 3 minutes to pellet stool particles and inhibitors bound to the InhibitEX matrix. Finally, 600 *μ*L of the supernatant was placed in the QiaCube purifier (Qiagen) for automated purification of the DNA. The QiaCube purifier was preloaded with proteinase K, AL buffer, ethanol, AW1 and AW2 buffers, and AE elution buffer. 

### 2.4. Polymerase Chain Reaction

Polymerase chain reaction (PCR) was performed in order to amplify the 16S rRNA genes. Each PCR reaction was performed in a total volume of 25 *μ*L, and the PCR conditions were as follows: HotFirePol 1.25 U (Solis Biodyne, Tartu, Estonia), B2 buffer 1x (SolisBiodyne), MgCl_2_ 2.5 mM (Solis Biodyne), dNTP 200 *μ*M (Termo Fisher scientific, Surrey, USA), forward primer 0.2 *μ*M, reverse primer 0.2 *μ*M. The amount of DNA template used was 5 ng. Amplicons were checked with 1.5% Agarose gel (80 V; 60 min).

The 16S rRNA genes were PCR amplified from each DNA extract using the GA universal cover-all 16S rRNA primers (Genetic Analysis, Oslo, Norway), providing a PCR product of approximately 1200 bp [19].

PCR amplification was carried out with an initial denaturation step at 95°C for 15 min, followed by 30 cycles consisting of denaturation for 30 sec at 95°C, annealing for 30 sec at 55°C, and elongation for 1 min 20 sec at 72°C. The reaction was completed with a final primer elongation step at 72°C for 7 min. 

### 2.5. Mixed Sequencing

16S rRNA genes form the stool samples were sequenced using the universally conserved primer U515FC30 [17]. Direct sequencing was performed for all the samples in order to obtain an overview of the bacteria composition and check for any indication that any of the dominant bacteria correlated with IBD. This operation was performed in replicates where both the PCR and the direct sequencing were repeated. In addition, 10 random samples were sequenced twice to function as technical replicates. 

Different dilution factors of the ExoI and SAP treated PCR products were used for the different samples. In order to decide the dilution factor, dilution series were performed based on the band strength of the agarose gel. This was done in order to obtain good sequencing signals, where the raw signals strength should be under 8000 relative fluorescent unit (rfu) (not saturated) and over 1000 rfu [18]. 

A multivariate curve resolution analysis (MCR) was carried out to resolve the mixed DNA sequence spectra into pure components and their relative amounts in each of the mixed DNA samples. This analysis included principal component analysis (PCA) in order to predict the number of components to be present in the dataset, followed by the MCR analysis to finally resolve the predicted number of components. This gives two outputs (i) the relative amount of each of the components in every sample of the dataset and (ii) the spectral information of each of the components. The spectral information was base called, and the components were aligned against entries in the Ribosomal Database Project II in order to classify them. 

### 2.6. Sequencing and Analyses of Clones

A total of 15 samples were selected for cloning. The cloning reaction and the transformation were performed using TOPO TA-cloning kit (Invitrogen) in accordance with the manufacturer recommendations for electrocompetent *E. coli*. 

Low quality sequences (poor signals and short sequences) were filtered out manually, and the forward and reverse sequencing reads that were of high quality were assembled using assemble sequences (default settings) in CLC Main Workbench v6.0.1. The assembled sequences that contained a high level of conflicting information were also filtered out. All the assembled sequences were aligned in CLC using default settings with* E. coli* U0096 being used as a reference. 

The sequences were further examined for chimeric artifacts using the chimeric sequence removal with chimera slayer in mothur (http://www.mothur.org/). The input in the chimera slayer was a fasta file of the filtered sequences in addition to a template file, and the outputs were potentially chimeric sequences based on the chimera slayer algorithm. The template reference set was obtained from Haas et al. [23]. 

The Ribosomal Database Project II Sequence Match and Classifier were used to classify the sequences to a taxonomical hierarchy. 

A phylogenetic tree was constructed based on the sequences from the clone libraries. The DNA sequences were aligned using the MUCSLE algorithm in CLC (default settings) before being imported as a fasta file into the online tool BioNJ which is a part of the online service Phylogeny.fr (http://www.phylogeny.fr/). The phylogenetic tree was constructed using the Kimura 2 parameters as substitution model and 1000 as bootstrap number. The tree was subsequently imported into the computer program Dendroscope (http://ab.inf.uni-tuebingen.de/software/dendroscope/) for editing. 

### 2.7. Probe Analyses

The 16S rRNA clone libraries were used to construct probes targeting the main clusters of bacteria. The DNA sequences in the clone libraries were first used to create a principal component (PC) plot by using the GA in-house-developed computer program PhyloMode (http://www.nofimamat.no/phylomode). Principal components analysis (PCA) is a method used for extracting a set of components that explain as much of the variability of a dataset as possible. The PhyloMode computer program is based on alignment-independent bilinear multivariate modeling (AIBIMM) [24]. The first step was to transform DNA sequence data into DNA *n*-mer frequencies. The *n*-mer frequency data was obtained by sliding a window of size *n*. A given pair of multimers can either be equal due to a common ancestor (homology) or equal due to mutational events (equal multimers with different evolutionary origin). A window size of *n* = 5 multimer was chosen as a trade-off between detecting phylogenetic signals (homologous multimer equalities) and avoiding base composition biases arising from nonhomologous multimer equalities [24]. The frequencies of the pentamers were counted and stored in a table. The multimer frequency data was normalized before being compressed into principal components (PCs) as previously described for the AIBIMM approach. The PCA model was exported as a “pcam” file for further use in TNTProbeTool. 

Before importing the sequences into PhyloMode as a file in FASTA format, all the sequences (with chimeras removed) were aligned in CLC. The sequences were cut at conserved regions at the beginning and end, giving them the same starting and ending point. 

The probe construction software TNTProbeTool was used for construction of the probes. TNTProbeTool is a GA in-house developed software for the design of single nucleotide primer extension (SNuPE) probes for analysis of microbial communities [19]. The TNTProbeTool has been developed to be able to find specific areas within the 16S rRNA gene and identify these as unique probes that can be used to identify a specific phyla, genera, family, or individual strains. The first step in the probe construction process was to define a set of multiple target and nontarget microbial DNA sequences in the PCA plot imported from the PhyloMode program. A matching region of eight nucleotides was chosen, and the labeling nucleotide was set as C. The next step was identification of probes that satisfied the criteria for target detection and nontarget exclusion, based on the combined criteria of hybridization and labeling. All probes were designed with minimum melting temperature (*T*_*m*_) of 60°C by the nearest-neighbor method for the target group, while the maximum *T*_*m*_ between probes and nontarget sequences was set at 30°C [19]. Finally, found probes were checked against nontarget sequences, and the probes that were not good enough were filtered out. The constructed probes were exported as a “fastagr” file.

The bacterial strain-specific probe was end-labeled with fluorescence dye TAMRA bound to a ddCTP (5-propagylamino-ddCTP-5/6 TAMARA) for detection using capillary electrophoresis. The designed probes were bound to the complementary 16S rRNA sequence of that particular bacterium or groups of bacteria, and ddCTP-TAMRA was then bound as a single nucleotide to the 3′ end of the probe. This reaction was done in a cyclic manner by thermocycling, and gave rise to free labeled probes in the solution. In a total volume of 10 *μ*L HOT Termipol DNA polymerase 2.5 U (Solis Biodyne), HOT Termipol buffer C 1x, MgCl_2_ 4 mM, ddCTP Tamra 0.4 *μ*M, designed probe 0.1 *μ*M, and 10x diluted ExoI and SAP treated template (2 *μ*L). The labeling reaction was carried out with an initial denaturation step at 95°C for 15 min, followed by five cycles consisting of denaturation for 20 sec at 96°C, and combined annealing and extension for 35 sec at 60°C. 

Before performing probe screening on all the samples, all the constructed probes were evaluated experimentally by cloned target sequences and nontarget sequences (both close to the target sequences and random sequences). Finally, suitable probes that satisfied the criteria for target sequences detection and exclusion of nontarget sequences were included in the screening.

All samples were hybridized with six probes in separate reactions. A universal 16S rRNA gene probe was also included to measure the total abundance of bacterial DNA in the samples (Table 2). After labeling, the samples were treated with 8 U SAP and incubated at 37°C for 1 hour and inactivated at 80°C for 15 min. Then 1 *μ*L of the SAP-treated and labeled probes were mixed with 9 *μ*L of Hi-Di formamide and 0.5 *μ*L GeneScan 120 Liz Size Standard (Applied Biosystems). The samples were incubated at 95°C for 5 min before being placed on ice. The samples were then loaded onto a 36 cm 3130xl capillary array in the ABI Genetic Analyzer 3130xl sequencer (Applied Biosystems), containing the performance optimized polymer 7 (POP-7, Applied Biosystems). Injection time was 16–22 s and the electrophoretic conditions were run time 180 s at 15000 V, run current 100 *μ*A, and 60°C run temperature. Data analysis was performed using the GeneMapper 4.0 software (Applied Biosystems). 

## 3. Results

### 3.1. Resolving Mixed Sequences into Pure Components

**Figure 2:**
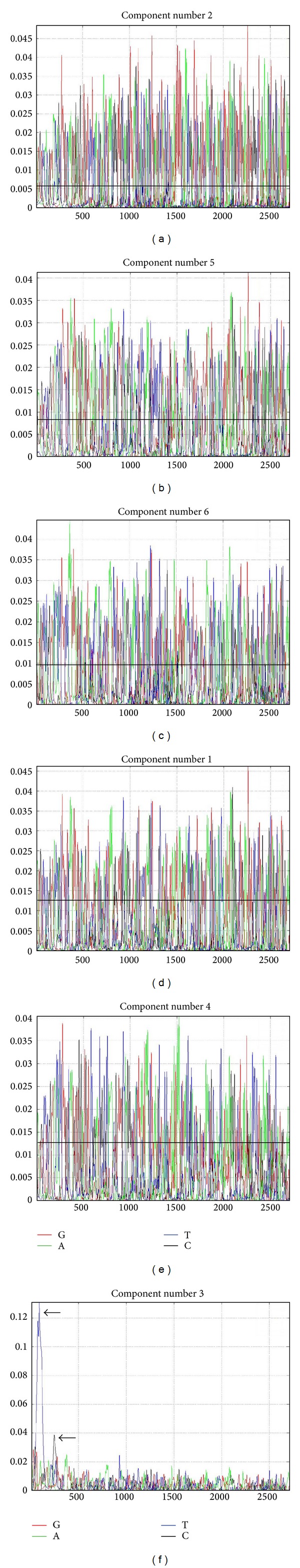
Spectra of the six components resolved by MCR analysis. Visual examination reveal components 2, 5, and 6 ((a)–(c)) to have well resolved spectra with low background sequences. Components 1 and 4 ((d) and (e)) also have well resolved spectra, although with somewhat higher background sequences than components 2, 5, and 6. Component 3 (f) has two high peaks (black arrows) and a poorly resolved spectrum.

The mixed sequences were resolved into six main components using MCR analysis. The spectra of the six components are presented in Figure 2. One of the components (component 3) was regarded as noise and excluded as it exhibited two high peaks and a poorly resolved spectrum. The other five components showed well-resolved spectra with nearly the same signal heights. There were, however, some variance in the signal height of the background sequences compared to the components and hence also in the purity of the components. A visual examination indicated that components 2, 5, and 6 had lower background sequences and better resolved spectra than did components 1 and 4. 

The base-called sequences of the five components with well-resolved spectra are shown in Table 3.

The components were classified using the Ribosomal Database Project II (RDP) Classifier (Table 4), which estimates the classification reliability using bootstrapping. Components 2, 5, and 6 were classified with relatively high bootstrap confidence estimates (above 85%) at the genus level, whereas for components 1 and 4, classification at the genus level gave low bootstrap confidence estimates (10% and 9% resp.). The confidence threshold for short sequences was set at 50%. And as a result, the components were classified as Clostridiales (Comp_1), *Escherichia/Shigella *(Comp_2), Clostridiales (Comp_4), *Faecalibacterium* (Comp_5), and *Bacteroides* (Comp_6).

The technical quality of the resolved components were evaluated both by analyses of sample replicates and comparison with the results from cloning and sequencing. Taken together, these results support a high technical quality and reliability. Details for the analyses and comparisons are shown in the Supplementary Material available online at http://dx.doi.org/10.1155/2013/636785.

### 3.2. MCR Clusters

**Figure 3:**
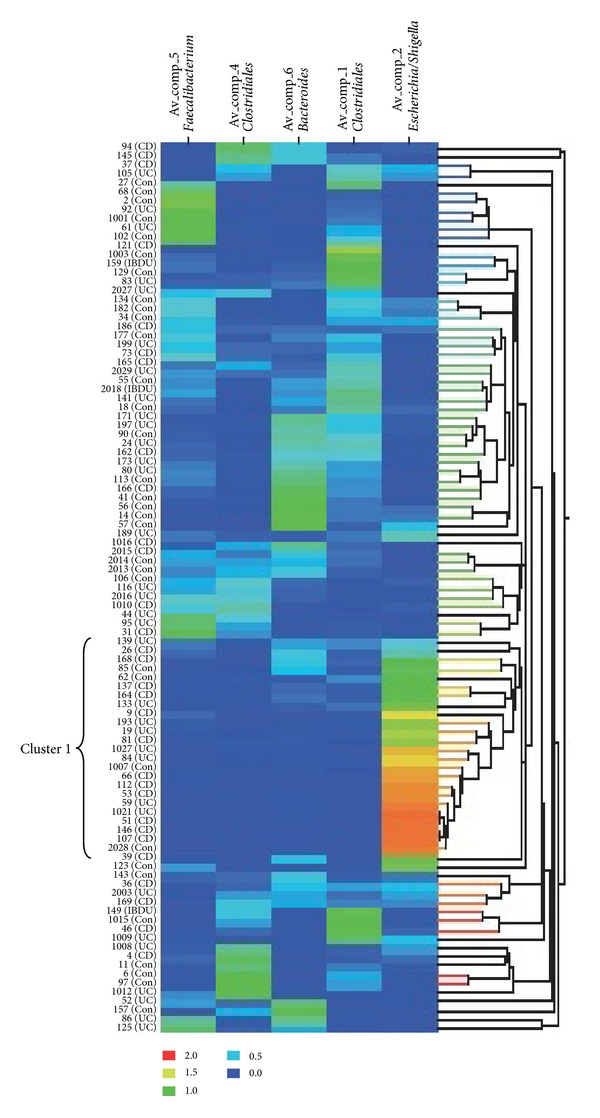
Data matrix of the amount of components 1, 2, 4, 5, and 6 in the stool samples. The graded colors indicate the abundance of a particular component in a sample. Red color indicates a high amount of the component, and blue color indicates a low amount of the component. To the left of the matrix, sample numbers are shown together with the diagnosis UC (Ulcerative colitis), CD (Crohn's disease), and Con (control).

The data matrix in Figure 3 summarizes the amount of each component in each sample.

One cluster of twenty-five samples (Cluster 1, Figure 3) has a very low diversity flora. There is mainly one component present; there are high amounts of Comp_2-*Escherichia/Shigella*, while the amounts of other components are low, or other components are not present at all. This cluster of samples consists of all disease states, though there is an overrepresentation of CD (52%) and UC (32%) compared to controls (16%). Most of the other samples show an overall mix of several components, and the data matrix does not reveal any apparent clustering of the different disease states. 

### 3.3. Comparison of the Average Amount of Components in the Different Disease States

**Figure 4:**
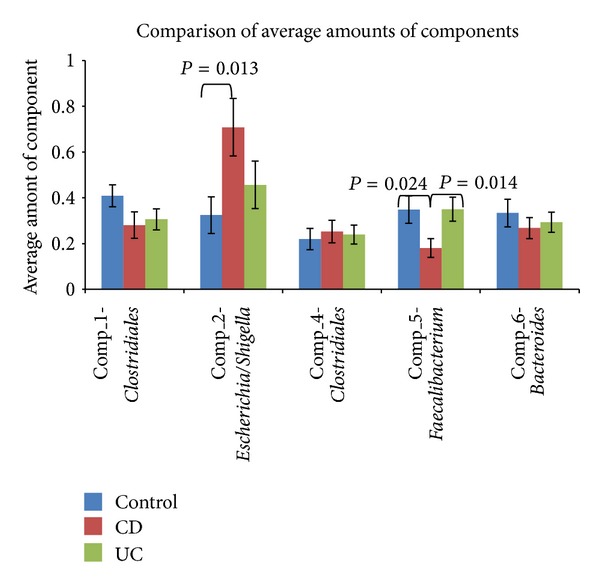
Comparison of the average amount of each component in the three disease states. The averages are calculated based on MCR analysis of stool samples from 33 subjects in the control group, 30 subjects diagnosed with CD (Crohn's Disease), and 33 subjects diagnosed with UC (Ulcerative Colitis). Standard error of arithmetic mean is shown. The significance of the differences between the averages of the three groups, control, CD, and UC, was tested using the *t*-test where the statistical significance was accepted at *P* < 0.05. Only the statistically significant *P* values are shown in the figure.

The average amount of each component was calculated for each of the three disease states in order to facilitate comparison between them. The averages are presented in Figure 4, and the most striking difference is for Comp_2-*Escherichia/Shigella*, where the CD average is high compared to both control and UC. Another considerable difference is the amount of Comp_5-*Faecalibacterium* present in the control and UC group, compared to the CD group. In addition, there is a slightly higher amount of Comp_1-Clostridiales in the control group compared to both CD and UC. 

In order to investigate whether the observed differences are statistically significant, a two-tail *t*-test for independent data was conducted. The amount of Comp_2-*Escherichia/Shigella* in CD patients was found to be statistically significantly higher (*P* = 0.013) than in controls, while the amount of Comp_5-*Faecalibacterium* was found to be significantly lower in CD patients than in both control subjects and UC patients (*P* = 0.024 and 0.014, resp.). The difference between the average amounts of Comp_1-Clostridiales in the control group and in CD and UC patients was not statistically significant at a 5% level (control versus CD; *P* = 0.097 and control versus UC; *P* = 0.129).

### 3.4. Comparison of the Average Signal Strength of the Probes in the Different Disease States

The probe identification and evaluation are presented in the Supplementary Information. Only one of the constructed probes did not satisfy the criteria of target detection and nontarget exclusion.

**Figure 5:**
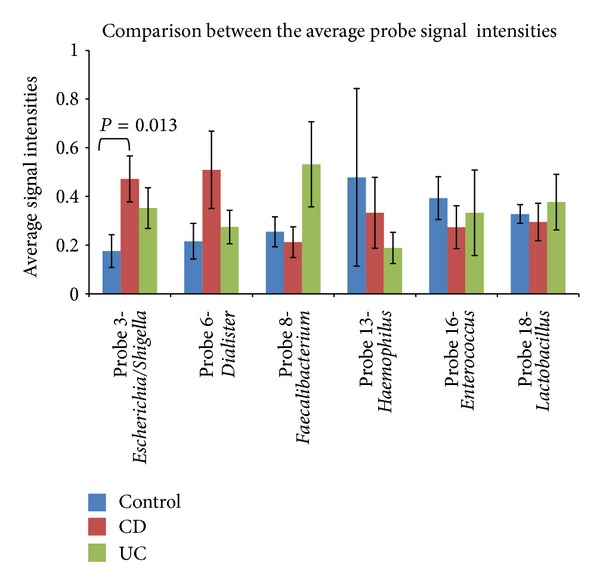
Comparison between the average amount of specific bacteria groups in the different disease states. The averages are calculated based on the height of the probe signals normalized by the signal height of the universal bacterial probe. Further, the averages are normalized to one because of Probe 18-*Lactobacillus* in particular, which gave much higher signals. There are 34 subjects in the control group, and the CD (Crohn's Disease) and UC (Ulcerative colitis) group have 30 and 33 subjects, respectively. Standard error of arithmetic mean is shown. The significance of the differences between the averages were tested using a *t*-test where the statistical significance was accepted at *P* < 0.05. Only the statistically significant *P*-value is shown in the figure.

 In order to make a comparison between the disease states, the average peak height for all the probes in the three disease states was calculated. The probe signals were normalized using signals from the universal probe, and the average of the ratios are presented in Figure 5. Because of considerable differences in absolute signal strength, all the signals were normalized to one. The most obvious difference is the signals of Probe 3-*Escherichia/Shigella *for both CD and UC compared to control. There is also a marked difference (*P* = 0.142 and 0.093, resp.) between UC and both CD and control for the signals of Probe 8-*Faecalibacterium*. In addition, CD has higher signals than both control and UC (*P* = 0.100 and 0.182, resp.) for Probe 6-*Dialister*. The average signal strength values were compared using a *t*-test and the only statistically significant (*P* = 0.013) difference was the higher amount of Probe 3-*Escherichia/Shigella* compared to controls. 

## 4. Discussion

The presence of *Escherichia/Shigella* was found to be significantly increased in CD patients compared to controls. The fact that both the MCR data and the probe screening data reveal a correlation of higher amounts of *Escherichia/Shigella* in patients with CD strongly supports this fact. The result, that is, increased numbers of *Escherichia/Shigella* in fecal samples from CD patients compared to control subjects is supported by several previous studies. Using a semiquantitative microbiological method, Giaffer et al. [25] found that patients with active CD had significantly higher total scores of *E. coli* compared to patients with quiescent disease, patients with UC, and healthy controls. Seksik et al. [26] further reported that enterobacteria were observed significantly more frequently in patients suffering from CD than in healthy subjects using dot blot hybridization. Using qRT-PCR and microarray approaches, Mondot et al. [27] revealed that *E. coli* is more represented in CD patients compared to controls. 

The predominant mucosa-associated bacterial communities in the colon differ significantly from those in feces [28, 29], and this is an important fact to recognize when studying the role of the endogenous microbiota in IBD [28]. An increased level of Proteobacteria (with *E. coli* being the most common phylotype) in CD patients compared to UC and controls was found in a study of tissue-associated intestinal microflora [30]. Also, an increased amount of Enterobacteriaceae has been found in CD mucosal biopsies [31]. Baumgart et al. [32] reported that the ileal mucosa of patients with CD involving the ileum were enriched in sequences of a novel group of invasive and potentially pathogenic *E. coli*, and that the number of *E. coli in situ* correlated with the severity of the disease. These mucosa-associated pathogenic *E. coli *are invasive and highly adherent to intestinal cells and are designated adherent-invasive *E. coli* (AIEC) [33, 34]. Darfeuille-Michaud et al. [35] found a high prevalence of AIEC in the ileal mucosa of patients suffering from CD. AIEC strains were found more frequently in early recurrent lesions after surgery, leading to the suggestion that AIEC could be involved in the initiation of the inflammatory process and not only secondary invaders. Sepehri et al. [36] characterized AIEC from IBD patients at first diagnosis, which suggests that they may have a role in the early stages of disease onset. The fact that AIEC is also detected in healthy mucosa [35], may indicate that the presence of these strains is in itself insufficient to cause disease. It has been suggested that AIEC may be opportunistic pathogens that have the ability to exploit the mucosal environment of a CD susceptible individual. Alternatively, the proliferation of these microorganisms may be a consequence of depletion of the normal flora [32]. AIEC may have the ability to exploit host defects in bacterial clearance and autophagy for survival and replication [37]. Furthermore, AIEC is able to initiate an inflammatory process by the induction of the first stages of cell aggregation leading to the formation of granulomatous structures [38] which is a histological characteristic of CD. Such granulomas are also associated with several infectious diseases involving among others *Salmonella *spp and *Shigella* spp (reviewed in Rolhion and Darfeuille-Michaud, [34]). 

In this study, *Faecalibacterium *were significantly less abundant in individuals with CD compared to both controls and individuals with UC when investigating the average of the MCR data. However, the probe for *Faecalibacterium* did not show significantly lower signals for the CD group compared to the control group or UC group. Although the average of the probe signals for the UC group showed a sizable difference compared to both the control group and the CD group, and the difference between UC and CD had a low *P* value; this was not statistically significant at the 5% level. There are, thus, some inconsistent results concerning *Faecalibacterium*. The abundance of *Faecalibacterium* seems, all the same, to be reduced in the CD group compared to both controls and the UC group. The Firmicutes phylum has previously been reported underrepresented in IBD, and in CD particularly. Manichanh et al. [39] reported a reduced diversity of Firmicutes, and the *Clostridium leptum* phylogenetic group in particular was reported to be less abundant in fecal samples from CD patients compared to those of healthy individuals. The *C. leptum* group contains numerous butyrate-producing bacteria. Butyrate is a major source of energy for colonic epithelial cells and inhibits inflammatory responses by decreasing proinflammatory cytokine expression via inhibition of NF-*κ*B activation in immune cells [40, 41]. Decreased butyrate levels could, thus, be implicated in the increased inflammatory state that occurs in IBD (reviewed in Fava and Danese, [42]). *Faecalibacterium prausnitzii* is a predominant species of the *C. leptum* group [43], and analysis has revealed that *F. prausnitzii *exhibit an anti-inflammatory effect and thus is important for the gut homeostasis [44]. A reduction of *Faecalibacterium* in fecal samples of patients with CD and an underrepresentation of the phylum Firmicutes, particularly *F. prausnitzii* in both active UC and CD patients as well as in infectious colitis patients has been reported [40, 45, 46]. Mondot et al. [27] also revealed that *F. prausnitzii* was more represented in fecal samples from healthy subjects compared to those of CD patients. One study, on the other hand, revealed a significant increase of *F. prausnitzii *at the time of diagnosis in pediatric CD suggesting a possibly more complex role for *F. prausnitzii *in CD pathogenesis. However, there may be important distinctions between adult and pediatric IBD [16]. 

The genus *Dialister* showed a higher abundance in CD patients in our study when comparing average probe signal strength although this was not significant at a 5% level when comparing the averages using* t*-test. In contradiction with this finding Joossens et al. [45] reported a decrease in *Dialister invisus* in patients with CD. This species is typically isolated from the oral cavity but has also been detected in samples of the normal gastrointestinal microbiota [47]. However, in the present study the probe for *Dialister* does not target the species *D. invisus* specifically which may be a possible explanation for the discrepancy. 

For the Bacteroides group we were not able to identify any significant correlations related to IBD. In the literature, however, there are conflicting evidence for Bacteroides. For a mouse model, it has been shown that Bacteroides species can induce colitis [48], while both significant [49, 50] and not significant [39] correlations have been identified for human cohorts. 

At a higher taxonomic level, the MCR analysis revealed one cluster of twenty-five samples consisting of all three disease states, although with an overrepresentation of CD and UC. These clusters showed low diversity flora with only one dominant component, Comp_2-*Escherichia/Shigella*. The low diversity flora was not expected, and in healthy individuals the abundance of Proteobacteria (including *E. coli*) is expected to be low [51]. However, the control group in this study are patients hospitalized with GI symptoms, and it can be disputed whether these patients can be characterized as healthy controls. Subjects in the control group may for instance be suffering from irritable bowel syndrome (IBS) which is a common intestinal disorder. The fecal microbiota has also been shown to be altered in patients suffering from IBS [52, 53], and discriminating IBS from IBD is a common clinical challenge [54]. The control patients in the present study all had symptoms without inflammation, probably also including IBS in several cases. Consequently, one strength of the study is its potential to differentiate between the characteristic of microbiota in inflammatory compared to noninflammatory states.

It is difficult to establish whether the altered microbiota composition observed in IBD patients is a cause or a consequence of the inflamed mucosa. The altered composition of microbiota may result from colonization by an enteric pathogen, from host-mediated inflammatory responses, or from both (reviewed in [55]). Infecting mice with *Salmonella enterica* serovar Typhimurium shows that this intestinal pathogen overcomes colonization resistance by inducing the host's inflammatory immune response and exploiting it for its own purpose and for promoting its own growth. An inflammatory response induced by *S. enterica *also alters the composition of the resident microflora. Other closely related proteobacteria, such as *E. coli*, is also believed to benefit from inflammation. The altered microbiota composition in IBD patients might, thus, not be the cause, but rather one of the many symptoms, of intestinal inflammation in IBD patients [56]. In a mouse model of gut infection, Lupp et al. [57] demonstrated that host-mediated inflammation in response to an infecting agent or genetic predisposition markedly alters the colonic microbial community. The resident colonic bacteria become significantly reduced whereas such an inflammation supports the growth of potentially pathogenic bacteria, particularly Enterobacteriaceae. These findings may suggest that the onset of an inflammatory response by the host could be the initiating factor in the dysregulation of the intestinal microbiota balance and cause of the persistent inflammatory state of IBD. An increased risk of developing IBD after an episode of acute gastroenteritis has also been indicated [58], which may lead to speculation that a bacterial infection-driven dysbiosis could lead to IBD in a predisposed individual [40]. Shifts in microbial populations are also associated with particular CD risk alleles, indicating that dysbiosis is not only a consequence of chronic disease [59]. Gophna et al. [30] found no significant difference in the flora between the ulcerated and nonulcerated tissues within the same individual suffering from CD and suggested that it is unlikely that inflammation is directly caused by a mucosa-associated pathogen. This is in agreement with another study reporting no qualitative difference between ulcerated and nonulcerated mucosa in CD patients [60]. In contradiction with this, Walker et al. [31] found differences in microbial community structure between inflamed and noninflamed mucosal sites. In UC patients, Zhang et al. [61] found a localized dysbiosis where lactobacilli and the Clostridium leptum subgroup were significantly different between the ulcerated and the nonulcerated regions of the mucosa-associated intestinal flora and that this may be related to UC. 

In conclusion, the evaluation of the fecal microbiota in newly diagnosed, untreated IBD patients and control subjects revealed significant changes in the fecal microbiota, whether causative of or responsive to disease.

## Supplementary Material

Suppl Table 1: R^2^ values illustrating the correlation between the two replicates of PCR amplification, direct sequencing and MCR analysis for one hundred stool samples.
Suppl Table 2. R^2^ values illustrating the correlation between the two replicates of the direct sequencing and MCR analysis for ten random PCR products.
Suppl Table 3: Composition of the clone library. The table summarizes the classification obtained in the Ribosomal Database Project II Classifier for all diagnosis groups. The classifications shown here are at the phylum, order and genus level with the confidence threshold set at 80%.
Suppl Figure 1: Graphical illustration of the reproducibility of the method. PCR amplification of the same extracted DNA from stool samples, direct sequencing and subsequent MCR analysis was performed in replicates for all hundred stool samples. The amount of the components in replicate 1 and 2 are plotted against each other for every one of the components in separate graphs. The R^2^ values are shown for all components.
Suppl Figure 2: Graphical illustration of the reproducibility of the direct sequencing and MCR analysis. Direct sequencing and MCR analysis were repeated on the same PCR product for ten samples. The amount of the components in replicate 1 and 2 are plotted against each other for every one of the components in separate graphs. The R^2^ values are shown for all components.
Suppl Figure 3: Phylogenetic distribution of the clone library. Neighbor joining tree showing clusters of five phyla; Firmicutes, Proteobacteria, Bacteroidetes, Actinobacteria and Verrucomicrobia. Multiple alignment was performed using the MUSCLE algorithm in CLC. The tree was constructed using the online tool BioNJ at Phylogeny.fr, and further visualized and edited using Dendroscope. Bootstrap values are based on 1000 replications. Sequences marked in red are covered by probes. The sequences are named according to hits from the Ribosomal Database Project Classifier, with a confidence threshold of 80%. In addition, the diagnoses CD (Crohn's disease), UC (Ulcerative colitis) and Con (control) as well as the ID of the samples from which the clones originated are indicated.
Suppl Figure 4: Comparison between the MCR-predicted relative amount of each component in the cloned samples and the relative amount of colonies were the component was detected. Amplified 16S rRNA gene sequences from 15 stool samples were selected for cloning based on high relative amounts of each of the five MCR predicted components.

## Figures and Tables

**Table 1:** Patient characteristics.

	CD	UC	IBDU	IBD total	Non-IBD
Total number	30	33	3	66	33
Median age	32.9	33.8	41.2	33.7	32.3
Min.–max.	20.1–52.7	16.1–60.1	34.9–52.2	16.1–60.1	19.1–55.1
Male	10	17	1	28	14
Female	20	16	2	38	19

**Table 2:** Sequence of the probes used in the project.

Probe	Sequence
Probe 3-*Escherichia/Shigella *	GCCTCAAGGGCACAAC
Probe 6-*Dialister *	AAGAACTCCGCATTTCTGC
Probe 8-*Faecalibacterium *	CGTAGTTAGCCGTCACTTC
Probe 13-*Haemophilus *	TCGCTTCCCTCTGTATACG
Probe 16-*Enterococcus *	CCCTCCAACACTTAGCA
Probe 18-*Lactobacillus *	CCTGTTTGCTACCCATACTTT
Universal probe	CGTATTACCGCGGCTGCTGGCA

**Table 3:** Base-called sequences of the five components that showed well resolved spectra obtained from the MCR analysis.

Component	Sequence
1 (Clostridiales)^1^	AGCGTTAGTCCGGATTTACTGGGTGTAAAGGGWGCGTAGGACGGWTGTGCAAGTCATG GAWGTGAAAGSCCCGGGGCTRAACCCCTGGYACTGCWTTTGGAAACTGTGAGACTAGG AGTGACWCGGAGYGGCTAASCGGAATTCCTAGTGTAGCGGTGAAATGCGTAGATATTAGG AGGAACACCAGTGGCGAAGGCGGCTTAGCTGGACTTGTAACTGACGRTGAGGCATCGAAA

2 (*Escherichia/Shigella*)^1^	AGCGTTAATCGGAATTACTGGGCGTAAAGCGCACGCAGGCGGTTTGTTAAGTCAGATGTG AAATCCCCGGGCTCAACCTGGGAACTGCATCTGATACTGGCAAGCTTGAGTCTCGTAGA GGGGGGTAGAATTCCAGGTGTAGCGGTGAAATGCGTAGAGATCTGGAGGAATACCGGTG GCGAAGGCGGCCCCCTGGACGAAGACTGACGCTCAGGTGCGAAA

4 (Clostridiales)^1^	AGGCGTTGTCCGGAATTATTGGGCGTAAAGSGCGCGCAGGCGGTTCCCTAAGTCCCTCTT AAAGTGGCGGGGCTTAACCCCGTGGATGGGAWGGAAACTGTGGAAGCTMGAGATTATC GGAAAGGAAAGTGGAATTCTCTATGTTYCGGTGGAAATGCGTAAAGAATTAGGAAGAAC AKCGGTTGGCGGAAGAGSCGACTTTCTGGAGCAAAACTGTAGCGCTCGTAGAGCSCCCAAA

5 (*Faecalibacterium*)^1^	AAGCGTTGTCCGATTACTGGGTGTAAGGGAGCGCAGGCGGAAGACAGTTGGAAGTGAAAC CATGGGCTCAACCCATGAATCTTGCTTTCAAAACRGMTTTTCTTGAYTWGTGCAAAGG GTAGAGTGGGAATTCCGGTTGTACCGTGGAATGCGTAATATCGGGAGGAACACCAGTGGC GAAGGCGGCRTACTGGGCACCAACTGACGCTGAGGCTCGAAA

6 (*Bacteroides*)^1^	AGCGTTATCCGGATTTATTGGGTTTAAAGGGAGCGTAGACTGGACTMTGTTAAGTCAGT TGTGAAAGTTTGCGGCTCAACCGTAAAATTGCAGTTGAWACTGGTGTCTTGAGTYCAGTW GAAGGCTYGGCGGAATTCGTGGTGTACGGTGAAATGCTTAATATCACGAAGAACRCCGAT TGCAAGGCAGCRTAGCTGAACTGAACTGACARTGATGCTCGAAA

^1^Classification of the components is done according to Table 3.

**Table 4:** The five well-resolved components from the MCR analysis of the results of the direct sequencing classified using the Classifier in Ribosomal Database Project II. Classification at the phylum, class, order, family, and genus levels are shown with the corresponding bootstrap confidence estimate.

	Phylum	Class	Order	Family	Genus
Comp_1	Firmicutes 84%	Clostridia 84%	Clostridiales* 84%	Lactinospiraceae 22%	*Lactinofactor* 10%
Comp_2	Proteobacteria 100%	Gammaproteobacteria 100%	Enterobacteriales 100%	Enterobacteriaceae 100%	Escherichia/Shigella* 85%
Comp_4	Frimicutes 77%	Clostridia 65%	Clostridiales* 64%	Incertae Sedis XI 15%	*Parvimonas* 9%
Comp_5	Firmicutes 98%	Clostridia 98%	Clostridiales 98%	Ruminococcaceae 97%	Faecalibacterium* 94%
Comp_6	Bacteroidetes 100%	Bacteroidia 99%	Bacteroidales 99%	Bacteroidaceae 91%	Bacteroides* 91%

*The cut-off value of the bootstrap confidence threshold was set at 50%. Comp_1 and Comp_4 were classified at the order level whereas the other components were classified at the genus level.
