# Supplementary material for: Dominant Fecal Microbiota in Newly Diagnosed Untreated Inflammatory Bowel Disease Patients
Source: Gastroenterol Res Pract. 2013 Nov 17;2013:636785. doi: 10.1155/2013/636785 (PMC3855989; doi:10.1155/2013/636785)
Supplement: Supplementary file 1 — Suppl Table 1: R2 values illustrating the correlation between the two replicates of PCR amplification, direct sequencing and MCR analysis for one hundred stool samples. Suppl Table 2. R2 values illustrating the correlation between the two replicates of the direct sequencing and MCR analysis for ten random PCR products. Suppl Table 3: Composition of the clone library. The table summarizes the classification obtained in the Ribosomal Database Project II Classifier for all diagnosis groups. The classifications shown here are at the phylum, order and genus level with the confidence threshold set at 80%. Suppl Figure 1: Graphical illustration of the reproducibility of the method. PCR amplification of the same extracted DNA from stool samples, direct sequencing and subsequent MCR analysis was performed in replicates for all hundred stool samples. The amount of the components in replicate 1 and 2 are plotted against each other for every one of the components in separate graphs. The R2 values are shown for all components. Suppl Figure 2: Graphical illustration of the reproducibility of the direct sequencing and MCR analysis. Direct sequencing and MCR analysis were repeated on the same PCR product for ten samples. The amount of the components in replicate 1 and 2 are plotted against each other for every one of the components in separate graphs. The R2 values are shown for all components. Suppl Figure 3: Phylogenetic distribution of the clone library. Neighbor joining tree showing clusters of five phyla; Firmicutes, Proteobacteria, Bacteroidetes, Actinobacteria and Verrucomicrobia. Multiple alignment was performed using the MUSCLE algorithm in CLC. The tree was constructed using the online tool BioNJ at Phylogeny.fr, and further visualized and edited using Dendroscope. Bootstrap values are based on 1000 replications. Sequences marked in red are covered by probes. The sequences are named according to hits from the Ribosomal Database Project Classifier, with a confidence thresh [file 636785.f1.pdf]

Supplementary information

Reproducibility of the MCR analysis

In order to evaluate the reproducibility of the method, the PCR amplification, direct sequencing and subsequent MCR analysis were performed in replicates. Graphical illustrations of the amount of the components in replicate 1 and 2 plotted against each other for every one of the components separately are shown in Suppl. Figure 1. The R^2 values obtained for each of the five components were between 0.85 and 0.95 (Suppl Table 1).

Suppl Table 1. R^2 values illustrating the correlation between the two replicates of PCR amplification, direct sequencing and MCR analysis for one hundred stool samples.

Component	R^2 value
Comp_1 – Clostridiales	0.860
Comp_2 – *Escherichia/Shigella*	0.949
Comp_4 – Clostridiales	0.930
Comp_5 – *Faecalibacterium*	0.891
Comp_6 - *Bacteroides*	0.849

In addition, to function as technical replicates, direct sequencing and MCR analysis were repeated on the same PCR products for ten random samples (Suppl Figure 2). The R^2 values for the components were found to be between 0.97 and 0.99 (Suppl Table 2), indicating very good correlation between the technical replicates.

Suppl Table 2. **R^2 values illustrating the correlation between the two replicates of the direct sequencing and MCR analysis for ten random PCR products.**

Component	R^2 value
Comp_1 – Clostridiales	0.966
Comp_2 – *Escherichia/Shigella*	0.999
Comp_4 – Clostridiales	0.999
Comp_5 – *Faecalibacterium*	0.998
Comp_6 - *Bacteroides*	0.980

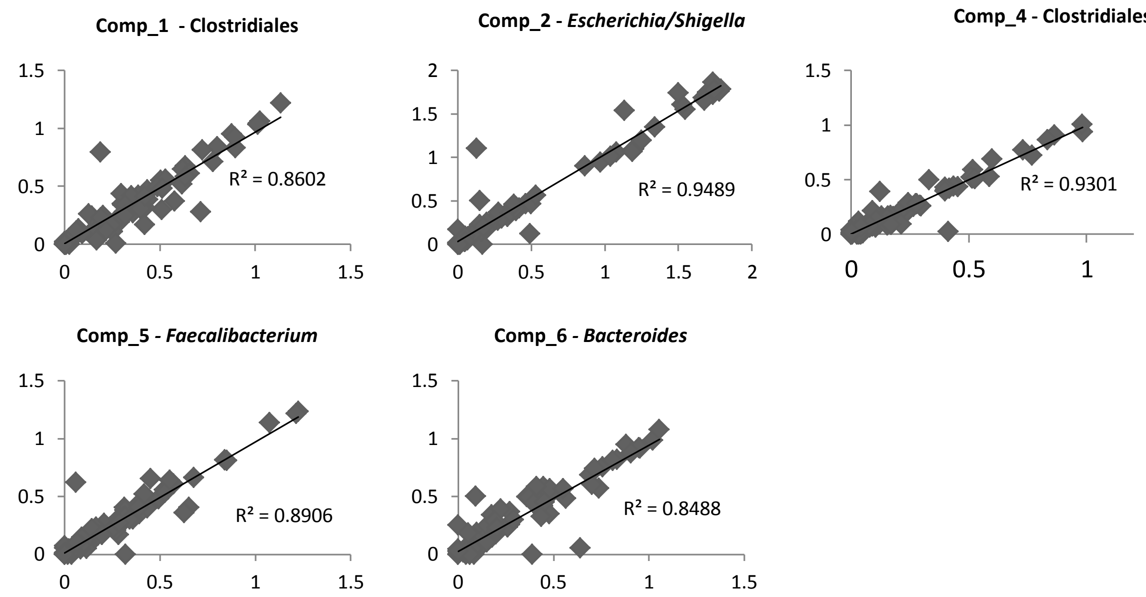

Suppl Figure 1. **Graphical illustration of the reproducibility of the method. PCR amplification of the same extracted DNA from stool samples, direct sequencing and subsequent MCR analysis was performed in replicates for all hundred stool samples. The amount of the components in replicate 1 and 2 are plotted against each other for every one of the components in separate graphs. The R^2 values are shown for all components.**

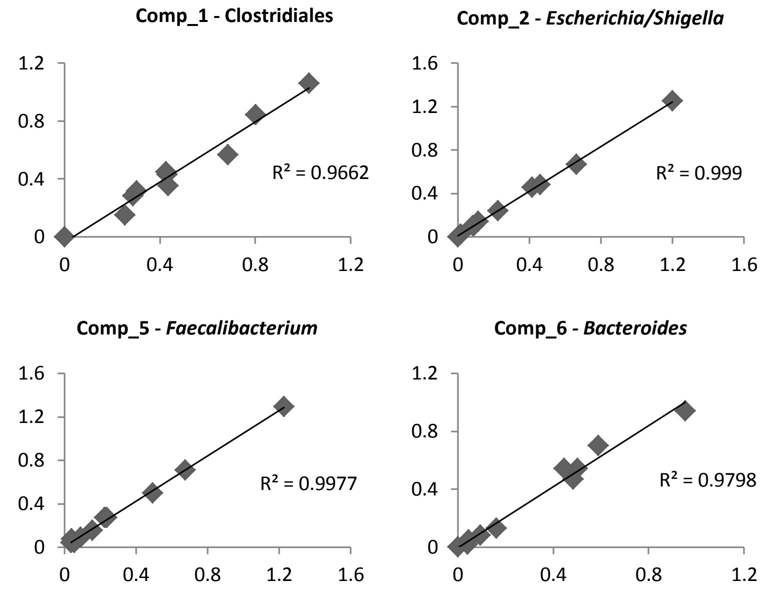

Suppl Figure 2. Graphical illustration of the reproducibility of the direct sequencing and MCR analysis. Direct sequencing and MCR analysis were repeated on the same PCR product for ten samples. The amount of the components in replicate 1 and 2 are plotted against each other for every one of the components in separate graphs. The R^2 values are shown for all components.

Composition of the clone library

The 575 clones with correct insertion of the 16S rRNA gene were sequenced using both forward and reverse primers. Out of these there were 235 clones in which both the forward and reverse sequence were of good quality. The assembled sequences were further analyzed with respect to length of the sequence as well as with respect to chimeric sequences. After filtering out short sequences (below 1000 bp) and the removal of chimeric sequences, a total of 190 sequences (57 from the control group, 50 from the CD group and 83 from the UC group) were included in the clone library.

The sequences were classified using Classifier in the RDP database, with a confidence threshold of 80%. The composition of the clone libraries are summarized in Suppl Table 3. The sequences were associated with five different phyla of Bacteria; Firmicutes (50% of clones), Proteobacteria (29%), Bacteroidetes (12%), Verrumicrobia (7%) and Actinobacteria (2%). The sequences in the clone library were used to create a phylogenetic tree, in order to visualize the relatedness of the sequences and how they were grouping. The phylogenetic tree of the clone library is presented in Suppl Figure 3, and the sequences group into the five different phyla as presented in Suppl Table

3. The most diverse phylum is the Firmicutes, which is composed of sequences belonging to 16 different genus in addition to some unclassified sequences. A total of 39% of the sequences in the Firmicutes phylum belongs to the genus *Dialister*, and 8% belongs to the *Faecalibacterium* genus. The sequences in the genus *Escherichia/Shigella* are closely related and constitute the whole Proteobacteria phyla, with the exception of a few *Haemophilus* sequences distinct from *Escherichia/Shigella*. The phylogenetic tree illustrates the relatedness of the sequences within the *Dialister* genus, *Faecalibacterium* genus and the *Escherichia/Shigella* genus, and these sequences were later used for constructing probes.

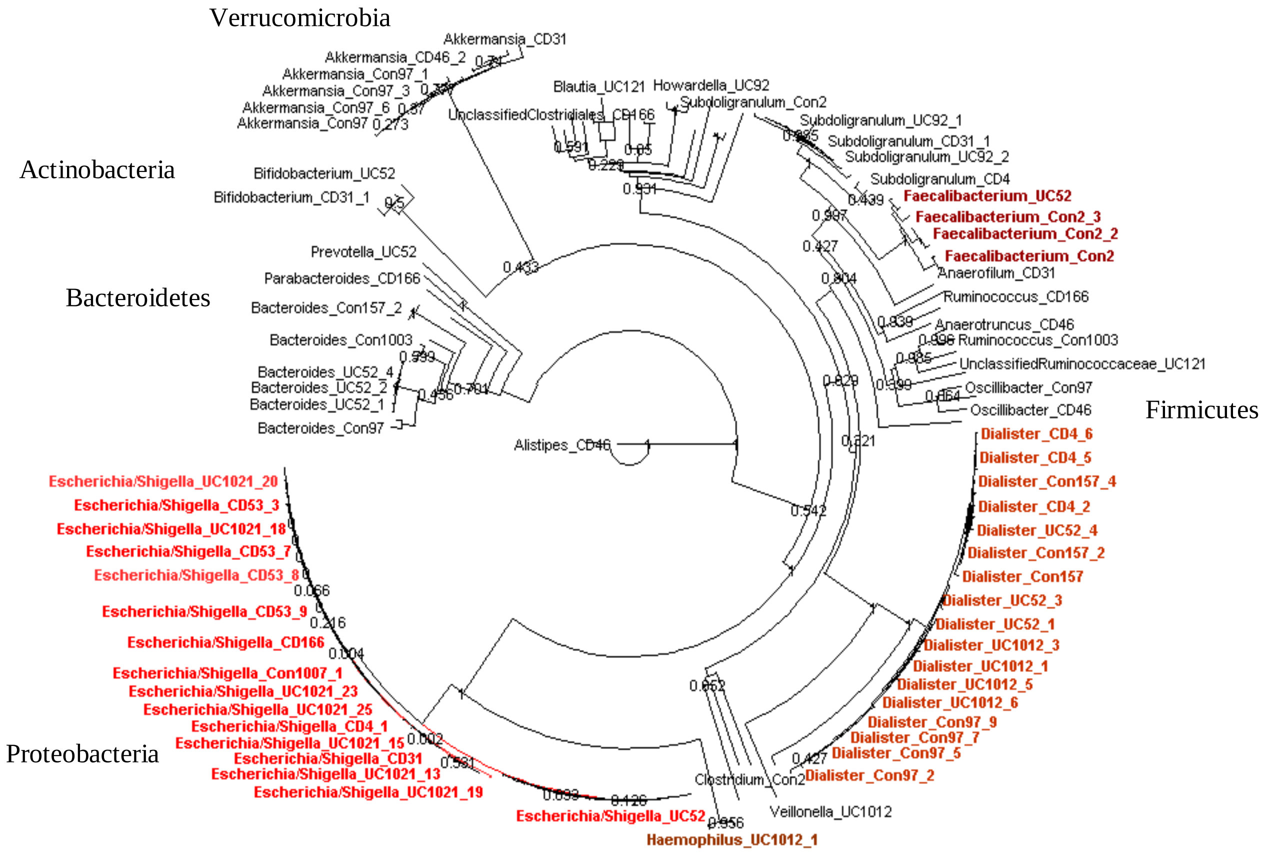

Suppl Figure 3. Phylogenetic distribution of the clone library. Neighbor joining tree showing clusters of five phyla; Firmicutes, Proteobacteria, Bacteroidetes, Actinobacteria and Verrucomicrobia. Multiple alignment was performed using the MUSCLE algorithm in CLC. The tree was constructed using the online tool BioNJ at Phylogeny.fr, and further visualized and edited using Dendroscope. Bootstrap values are based on 1000 replications. Sequences marked in red are covered by probes. The sequences are named according to hits from the Ribosomal Database Project Classifier, with a confidence threshold of 80%. In addition, the diagnoses CD (Crohn's disease), UC (Ulcerative colitis) and Con (control) as well as the ID of the samples from which the clones originated are indicated.

Suppl Table 3. Composition of the clone library. The table summarizes the classification obtained in the Ribosomal Database Project II Classifier for all diagnosis groups. The classifications shown here are at the phylum, order and genus level with the confidence threshold set at 80%.

Phylum	Order	Genus	Control	CD	UC
**Frimicutes (95)**	Clostridiales (93)	Activibrio	1		
		Anaerofilum		1	
		Anaerotruncus		1	
		Blautia		1	2
		Clostridium	1		
		Dialister	15	8	14
		Dorea		1	
		Faecalibacterium	4	1	3
		Howardella			1
		Oscillibacter	2	1	
		Roseburia	3		1
		Ruminococcus	2	1	
		Subdoligranulum	2	5	7
		Veillonella			1
		Unclassified	6	2	6
	Lactobacilli (1)	Granulicatella		1	
	Erysipelotrichales (1)	Holdemania			1
		Total	36	23	36
**Bacteroidetes (22)**	Bacteroidales (22)	Alistipes	2	1	
		Bacteroides	6	1	9
		Parabacteroides		1	
		Prevotella	1		1
		Total	9	3	10
**Proteobacteria (55)**	Enterobacteriales (52)	Escherichia/Shigella	3	18	31
	Pasteurellales (3)	Haemophilus			3
		Total	3	18	34
**Verrucomicrobia (14)**	Verrucomicrobiales (14)	Akkermansia	9	4	1
**Actinobacteria (4)**	Actinobacteridae (4)	Bifidobacterium		2	2
TOTAL			57	50	83

Comparison of MCR-predicted components and clone library

Comparison between the MCR-predicted relative amount of components in the cloned samples, and the number of colonies where the component was detected, reveals a relatively good correspondence between the two. Suppl Figure 4 compares the MCR-predicted relative amount of the component in the cloned samples and the relative amount of colonies where this component was actually detected. In the samples that were cloned because of high amounts of Comp_1 - Clostridiales, Comp_2 – *Escherichia/Shigella* and Comp_4 – Clostridiales, there were a high overall amount of colonies where these components were present. The samples that were cloned based on their high relative amount of Comp_5 – *Faecalibacterium* and Comp_6 – *Bacteroides*, on the other hand, had lower amounts of colonies present than what was predicted by the MCR analysis. Two samples that were cloned based on a high amount of Comp_5 – *Faecalibacterium*, and one sample cloned based on a high amount of Comp_6 – *Bacteroides*, yielded no colonies with these components present at all. However, the numbers of colonies obtained from some of these samples were low.

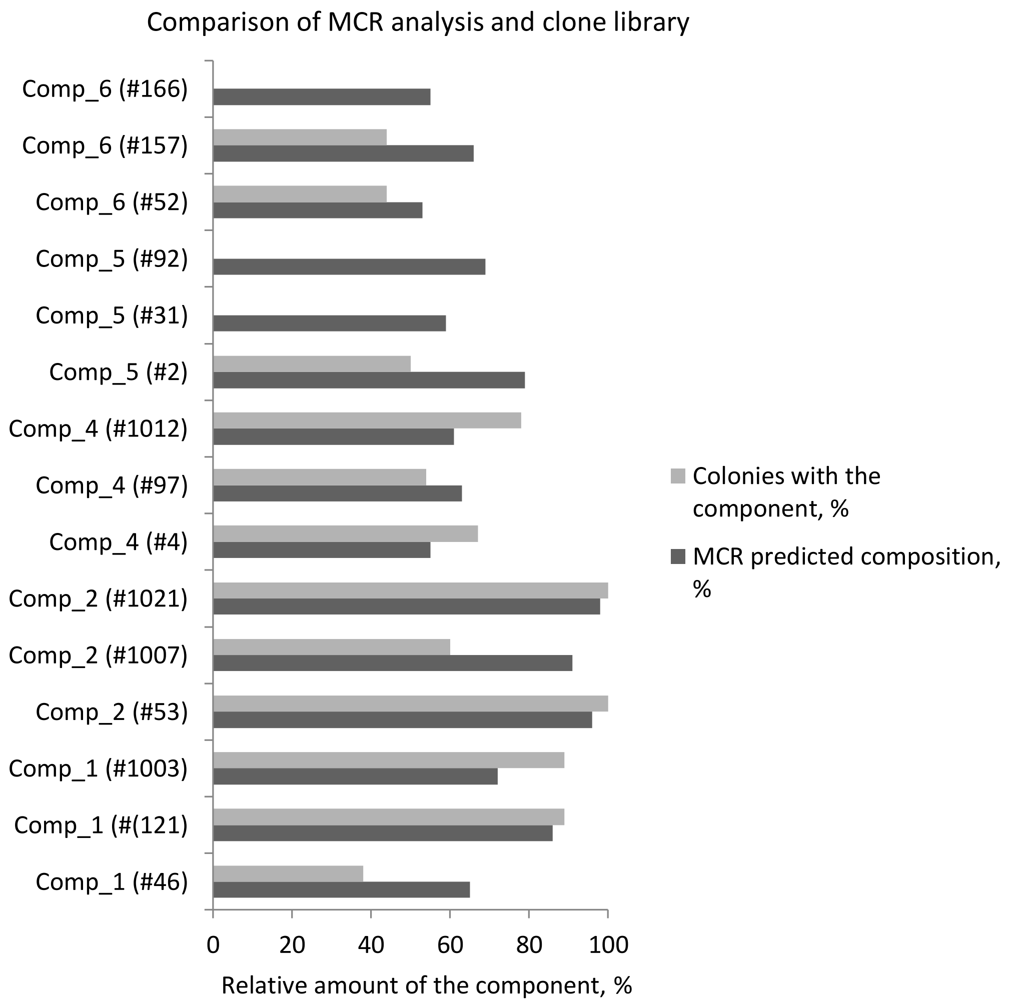

Suppl Figure 4. Comparison between the MCR-predicted relative amount of each component in the cloned samples and the relative amount of colonies were the component was detected. Amplified 16S rRNA gene sequences from 15 stool samples were selected for cloning based on high relative amounts of each of the five MCR predicted components.

Probe construction and evaluation

The 16S rRNA gene sequences were arranged into PCA plots based on their multimer-frequencies by the computer program PhyloMode. These PCA plots show clusters of *Dialister*, *Faecalibacterium* and *Escherichia/Shigella*. To find probes for these groups of bacteria, the clusters of these sequences were marked as target while the other sequences were marked as non-target. The probes were further constructed based on the criteria described in Materials and Methods.

The constructed probes were exported as “fastagr” files and subsequently checked against the clone library again to obtain probe statistics.

Probes with no target or too many unclassified sequences (>25%) were rejected, and from the remaining probes three of the best probe candidates from each bacteria group were chosen for experimental evaluation.

A total of nine probes (three probes for each of three groups of bacteria; *Dialister*, *Faecalibacterium* and *Escherichia/Shigella*) were selected for experimental evaluation. For each probe, two target sequences and three non-target sequences (one close to the target and two random) were tested as a template .The probes that were selected as good probes had high signal values for the target bacteria sequence (target detection) and no signal for the non-target bacteria sequence (non-target exclusion). All the evaluated probes except one satisfied the criteria for target detection and non-target exclusion.
